# Conditioned medium produced by fibroblasts cultured in low oxygen pressure allows the formation of highly structured capillary-like networks in fibrin gels

**DOI:** 10.1038/s41598-020-66145-z

**Published:** 2020-06-09

**Authors:** Christophe Caneparo, Clément Baratange, Stéphane Chabaud, Stéphane Bolduc

**Affiliations:** 1 Centre LOEX de l’Université Laval, Génie tissulaire et régénération, Centre de Recherche du CHU de Québec-Université Laval, Regenerative Medicine Division, Québec, QC Canada; 2 Programme Analyses Biologiques et Biochimiques, Institut Universitaire de Technologie de Laval, Laval, France; 3 Department of Surgery, Faculty of Medicine, Université Laval, Quebec, QC Canada; 4 Present Address: Université de Reims Champagne-Ardenne, UMR-I 02 SEBIO, Unité Stress Environnementaux et BIOsurveillance des milieux aquatiques, UFR Sciences Exactes et Naturelles, Campus du Moulin de la Housse, Reims, France

**Keywords:** Biomaterials, Tissue engineering

## Abstract

Tissue engineering is an emerging and promising concept to replace or cure failing organs, but its clinical translation currently encounters issues due to the inability to quickly produce inexpensive thick tissues, which are necessary for many applications. To circumvent this problem, we postulate that cells secrete the optimal cocktail required to promote angiogenesis when they are placed in physiological conditions where their oxygen supply is reduced. Thus, dermal fibroblasts were cultivated under hypoxia (2% O_2_) to condition their cell culture medium. The potential of this conditioned medium was tested for human umbilical vein endothelial cell proliferation and for their ability to form capillary-like networks into fibrin gels. The medium conditioned by dermal fibroblasts under hypoxic conditions (DF-Hx) induced a more significant proliferation of endothelial cells compared to medium conditioned by dermal fibroblasts under normoxic conditions (DF-Nx). In essence, doubling time for endothelial cells in DF-Hx was reduced by 10.4% compared to DF-Nx after 1 week of conditioning, and by 20.3% after 2 weeks. The DF-Hx allowed the formation of more extended and more structured capillary-like networks than DF-Nx or commercially available medium, paving the way to further refinements.

## Introduction

The life expectancy of humans is, at least and in part, related to their access to high-quality health care and, therefore, mostly dependent on technological innovations in this field. Recently, people’s lifestyles have evolved in developed countries to become increasingly sedentary. This evolution has resulted in a significant increase in chronic diseases, which could eventually require organ replacement^1^. However, replacement organs must meet increasingly stringent regulatory standards in order to protect patients^2^.

Consequently, the availability of graftable organs is no longer sufficient to meet the demand, and new solutions are required. Almost 30 years ago, Langer and Vacanti reported on a new concept, and tissue engineering emerged^3^. It allows the *in vitro* reconstruction of organs to be grafted in patients. This technology has evolved significantly to generate products that meet the required criteria. First, mainly consisting of inert biomaterials, the reconstructed tissues have increasingly integrated host cells and functionalization of the surfaces of these biomaterials to bring them as close as possible to the native tissues^4^. To provide substitute organs, tissue engineering has also led to the emergence of particularly promising three-dimensional study models (e.g.^5,6^). However, there are still many obstacles to overcome, especially for thick tissue grafts.

Nowadays, the graft take of thick engineered tissues, cellularized or not, remains a challenge due to the delay of reperfusion of the graft, which rapidly becomes ischemic, necrotic and generates conflicting signals^7,8^. The wound bed is the source of host vessels, which will colonize the engineered tissue through angiogenesis. The endothelialization of the engineered tissue before grafting represents a strategy of choice by reducing the time required to provide oxygen and nutrients to the cells. Nevertheless, the extracellular matrix (ECM) is essential for the formation of a microvascular network^9^. Most of the biomaterials could not sustain angiogenesis by themselves and required that the scaffold was seeded by ECM-producing cells before or at the same time than endothelial cells seeding^10–12^. The expansion of these cells, and their subsequent *in vitro* culture into the scaffold, often required specific cell culture medium containing several recombinant proteins (e.g., endothelial cell growth medium from PromoCell or Cell Application Inc. or EGM-2 from Lonza). Unfortunately, these molecules are expensive, and their potential use by most laboratories is limited. Even with the use of this specific cell culture media, endothelialization of the scaffold could be inadequate or immature. There is a need to find an alternative medium, less expensive and more effective than the one currently commercially available.

Angiogenesis is the physiological process that allows the formation of new blood vessels from pre-existing ones. This process is very active during development but becomes limited to few physiological (e.g. menstrual cycle, placenta formation, wound healing) or pathological (e.g. cancer) conditions at the adult age. When cells lack oxygen, i.e. are under hypoxic conditions, they release factors to trigger angiogenesis^13^. The vascular network answers quickly to this proangiogenic signal by promoting the reorganization of blood vessels, with the emergence of a tip cell leading the migration of its neighbouring and proliferating endothelial cells until blood flow is re-established for the cells in need. A process of maturation and regression of the vascular network assures that the adequate amount of oxygen and nutrients will be available.

We postulate that cultivating normal dermis fibroblasts under hypoxic conditions (i.e. 2% of O_2_ instead of 20%) will allow the release, in the cell culture medium, of the adequate factors required to recapitulate *in vitro* the different steps of angiogenesis. This conditioned medium can be subsequently used to cultivate endothelial cells and produced a pre-endothelialized scaffold. In this article, we are testing the potential of such a conditioned medium for the formation of a microvascular network in fibrin gels, and we are examining the expression of several pro-angiogenic factors in DF-conditioned medium under hypoxic vs normoxic conditions.

## Results

### Fibroblast-conditioned medium sustains greater human umbilical vein endothelial cell growth compared to fresh medium

**Figure 1:**
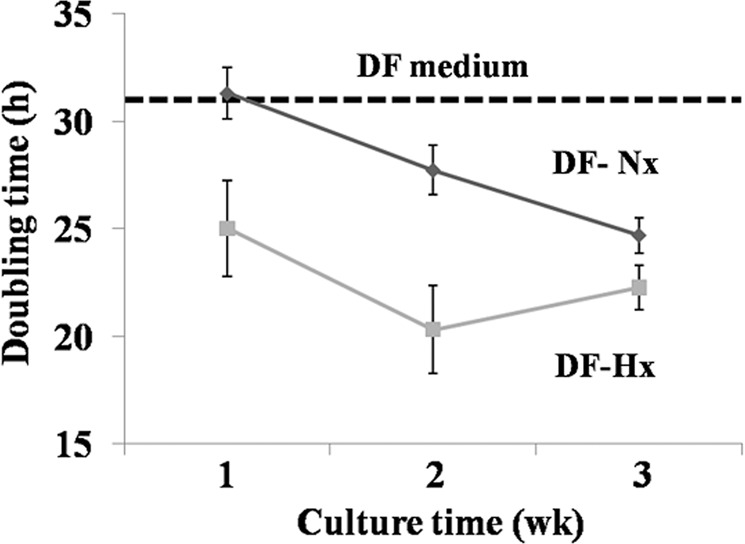
Medium conditioned by dermal fibroblasts under hypoxic conditions increased the proliferation of human umbilical vein endothelial cells. Doubling time was calculated from growth curves determined after cell counts for unconditioned medium (DF medium, black dash line), medium conditioned by dermal fibroblasts under normoxic conditions (DF-Nx medium, solid black line) and medium conditioned by dermal fibroblasts under hypoxic conditions (DF-Hx medium, solid grey line). The periods of conditioning were 1, 2 or 3 weeks. Results are presented as average +/− standard deviation.

HUVEC doubling time was calculated after the cells were cultivated in the presence of DF, DF-Nx or DF-Hx medium. For the last two conditioned media groups, the media were conditioned 1, 2 or 3 weeks after DF reached confluence. Doubling time was obtained by using DF-Hx (25.0 h, 20.3 h and 22.6 h for 1, 2 and 3-week of conditioning, respectively) were significantly reduced compared to the conditioned DF-Nx (27.9 h, 25.5 h, and 24.7 h for 1, 2 and 3-week of conditioning, respectively) with a p = 0.026, 0.014 and 0.035 respectively (Fig. 1).

### Conditioned medium from fibroblast cultivated in the hypoxic atmosphere sustains a more significant capillary-like network development than conditioned medium from fibroblast cultivated in normoxic atmosphere or endothelial cell growth commercial medium

**Figure 2:**
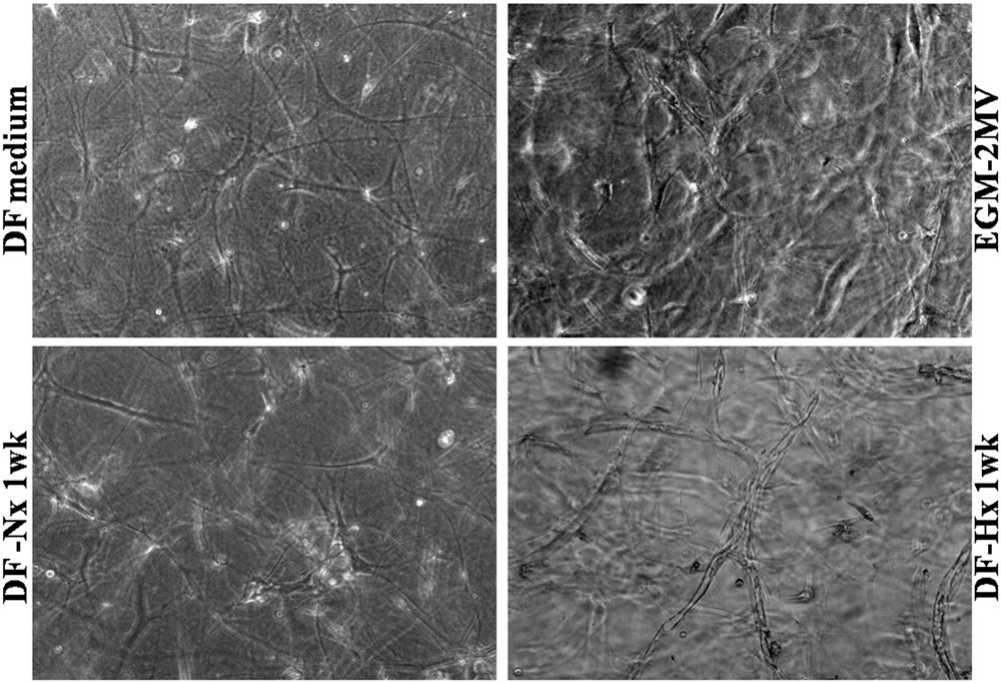
Medium conditioned by dermal fibroblasts under hypoxic conditions allowed the formation of a highly structured capillary-like network by human umbilical vein endothelial cells. Representative microscope photographs taken on gels after human umbilical vein endothelial cells were cultivated with irradiated fibroblasts seeded in fibrin gels with unconditioned medium (DF medium, left upper panel), commercially available medium EGM-2MV (EGM-2MV, right upper panel), medium conditioned during 1 week by dermal fibroblasts under normoxic conditions (DF-Nx medium, left lower panel) and medium conditioned during 1 week by dermal fibroblasts under hypoxic conditions (DF-Hx medium, right lower panel). Whereas no structures could be observed for DF medium and DF-Nx, capillary-like structures were visible for EGM-2MV and DF-Hx conditions. The network was more elaborated with DF-Hx. Tubulogenesis could be observed with the inner vacuole, which formed the lumen.

The potential of DF-Hx was then compared to DF medium, DF-Nx and commercially available medium specific for endothelial cell culture, EGM-2MV. Contrarily to conditions where DF medium (Fig. 2, left upper panel) and DF-Nx (Fig. 2, left lower panel) were used to cultivate fibrin gels, where no capillary-like network could be observed after 14 days, fibrin gels cultivated using EGM-2MV (Fig. 2, right upper panel) presented several nascent capillary-like structures. Nevertheless, conditions using DF-Hx (Fig. 2, right lower panel) presented very well organized capillary-like structures where the fusion of vacuoles of the interconnected HUVEC formed the lumen of the microvessels. The number of capillary-like structures in this latter condition was also higher (Fig. 2).

**Figure 3:**
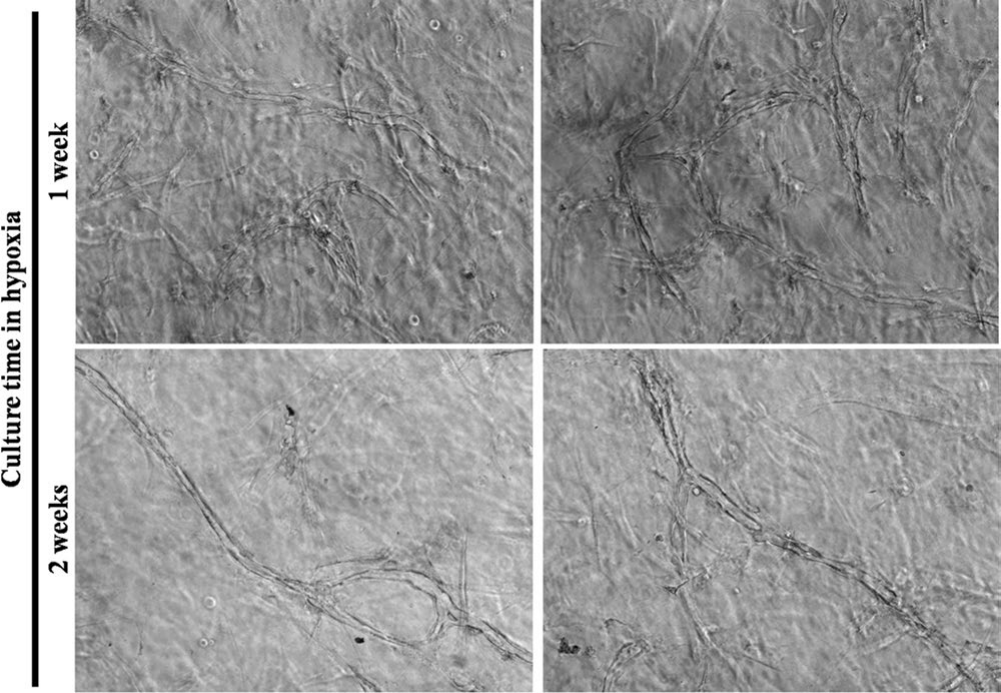
Increasing the duration of the conditioning period did not increase the number of capillary-like structures. Representative microscope photographs taken on gels after human umbilical vein endothelial cells were cultivated with irradiated fibroblasts seeded in fibrin gels with medium conditioned by dermal fibroblasts under hypoxic conditions during 1 week (upper panels) or 2 weeks (lower panels). Despite a more extended period of conditioning, the 2 weeks-condition did not promote a higher number of structures. It is possible that fewer structures were present in this latter condition, but the 3D context did not allow a precise determination of such parameters using brightfield microscopy. Once again, tubulogenesis was visible with the inner vacuole forming the lumen.

Increasing culture time between the time the cells reached confluence and the time of harvesting of conditioned media from 1 week (Fig. 3, upper panels) to 2 weeks (Fig. 3, lower panels) did not change the maturation level of the observed capillary-like structures but seemed to slightly reduce their number (Fig. 3).

**Figure 4:**
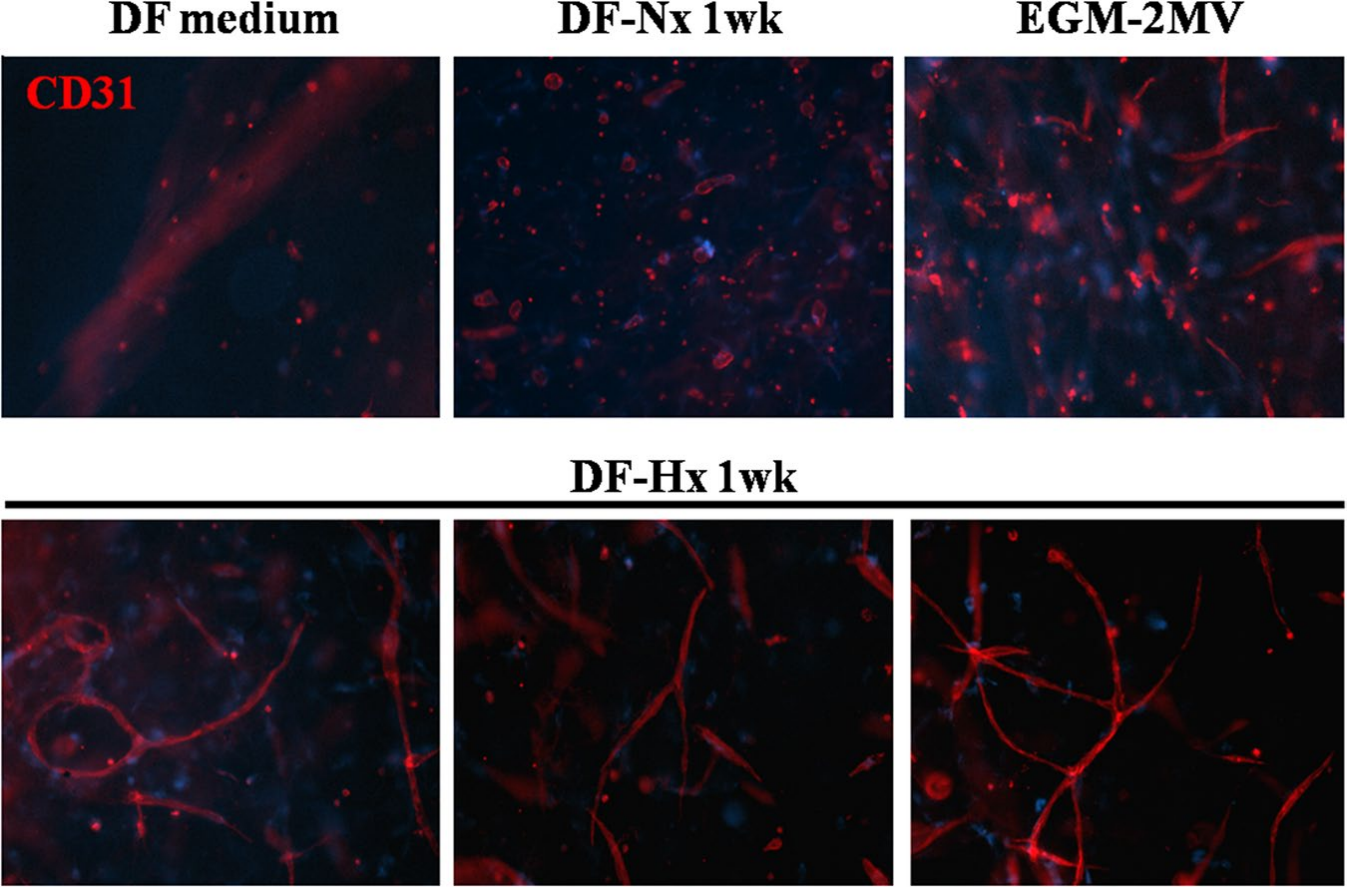
Medium conditioned by dermal fibroblasts under hypoxic conditions allowed the formation of a CD-31 positive, highly structured capillary-like network by human umbilical vein endothelial cells. Representative fluorescent microscope photographs taken on CD-31 immuno-labelled gels after human umbilical vein endothelial cells were cultivated with irradiated fibroblasts seeded in fibrin gels with unconditioned medium (DF medium, left upper panel), medium conditioned during 1 week by dermal fibroblasts under normoxic conditions (DF-Nx medium, central upper panel), commercially available medium EGM-2MV (EGM-2MV, right upper panel), and medium conditioned during 1 week by dermal fibroblasts under hypoxic conditions (DF-Hx medium, lower panels). No structures could be seen in DF medium condition and few ones in the DF-Nx condition. EGM-2MV condition showed several structures but with a reduced degree of maturation compared to what was obtained using DF-Hx.

As brightfield microscopy cannot confirm that the observed structures are formed by the HUVEC, immunofluorescent (IF) labelling was performed using antibodies raised against CD-31. In our model, i.e. fibrin gels seeded with irradiated DF and HUVEC, DFs are negative for this marker, and the signal can only originate from HUVEC. The structure previously observed was positive for CD-31 and the capillary-like structures derived from HUVEC (Fig. 4).

**Figure 5:**
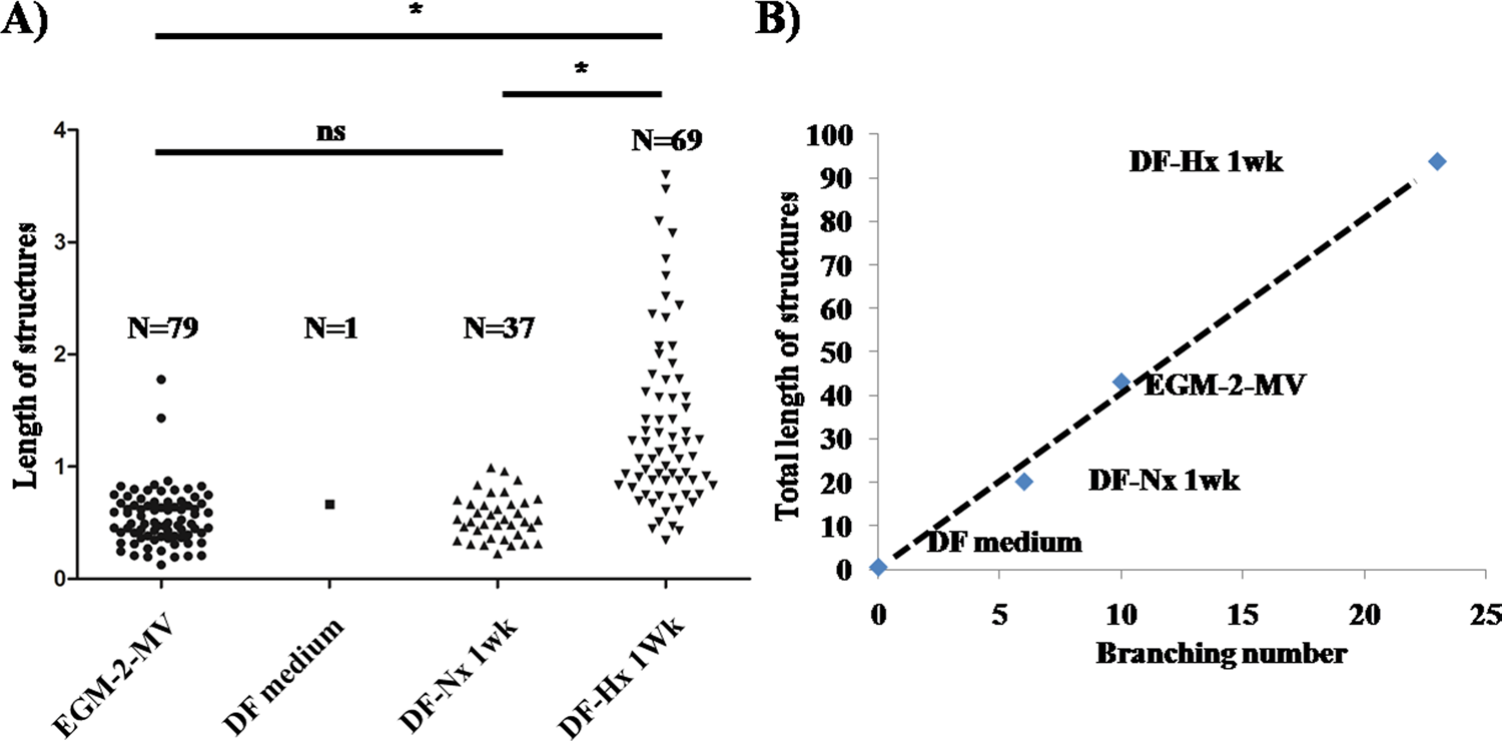
Quantification of length and branching number of capillary-like structures observed in fibrin gels. the CD-31 immuno-labelling, length and branching number of the capillary-like structures were measured for conditions using commercially available medium EGM-2MV (EGM-2MV), unconditioned medium (DF medium), medium conditioned during 1 week by dermal fibroblasts under normoxic conditions (DF-Nx), and medium conditioned during 1 week by dermal fibroblasts under hypoxic conditions (DF-Hx). (**A**) Quantification of the length of the structures. N is the number of observed structures in the central field of 3 distinct gels. The length was expressed as the value provided by ImageJ software. (**B**) Comparison of the total length of capillary-like structures and their branching number.

The number and lengths of the capillary-like structures were determined from the CD-31 IF. The length of a structure was measured between two branches. Only one structure was observed for DF medium cultivated fibrin gels, which probably consisted of artefact adjacent cells. Fibrin gels cultivated using DF-Nx presented few short length structures. Conditions using EGM-2MV and DF-Hx showed a roughly similar number of structures, but their average length was significantly higher in DF-Hx condition (1.358, p < 10^−6^) than all other conditions (0.546 and 0.548 for EGM-2MV and DF-Nx, respectively), not significantly different between each other with a p = 0.96 (Fig. 5A). The branching number was also compared to the total length of structures. Not surprisingly, the DF medium did not present branching. Increasing length and branching were found for DF-Nx, EGM-2MV, and DF-Hx. This last condition showed a twofold increase in total length and branching number of capillary-like structures compared to the commercially available medium, and a fivefold increase in total length and branching number than the DF-Nx condition (Fig. 5B).

### Comparison of angiogenic mediators’ profiles of conditioned medium from fibroblast cultivated in hypoxic atmosphere vs conditioned medium from fibroblast cultivated in the normoxic atmosphere

**Figure 6:**
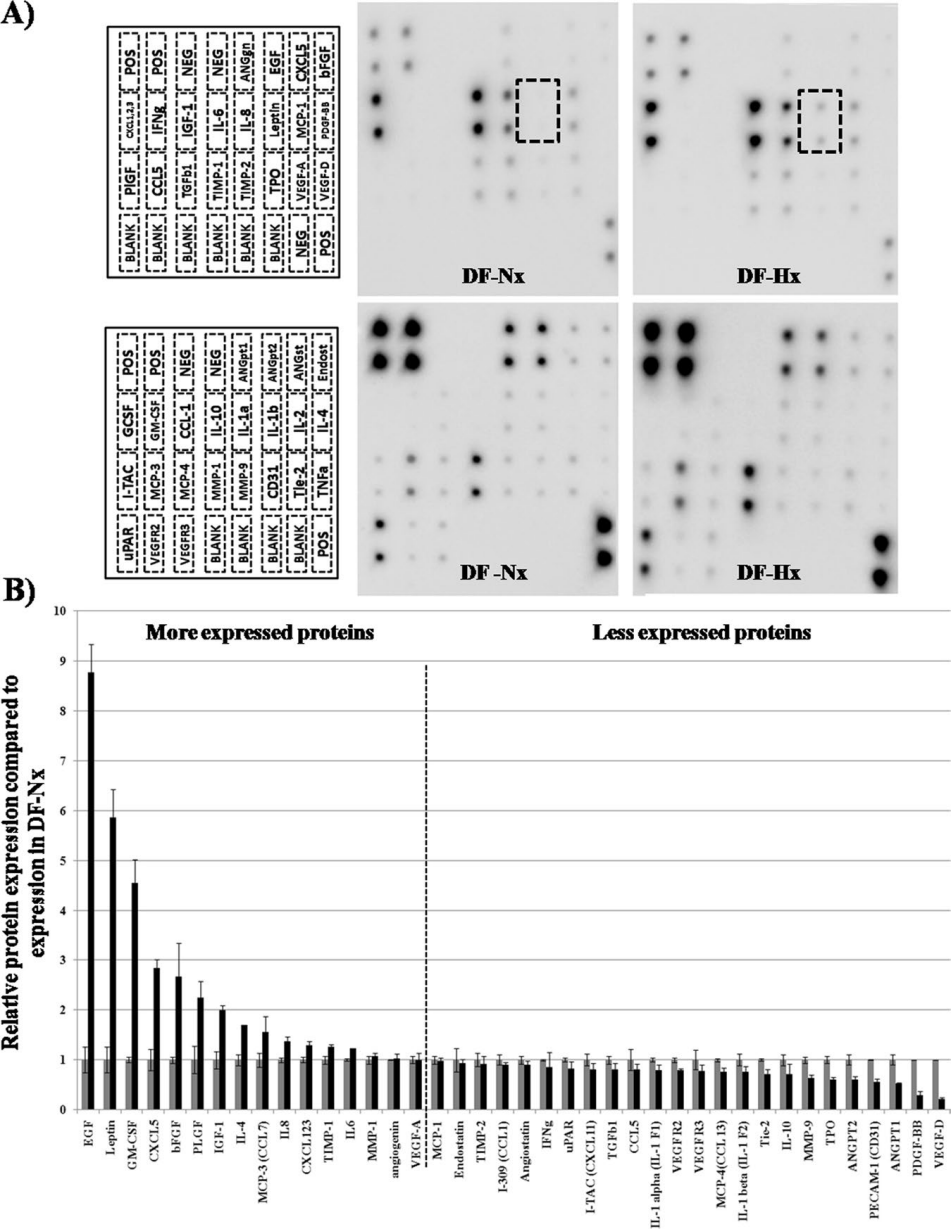
Determination of the angiogenic profile of DF-Nx medium (1 week) and DF-Hx medium (1 week) using a commercially available macro-array. The medium conditioned during 1 week by dermal fibroblasts under normoxic conditions (DF-Nx), and medium conditioned during 1 week by dermal fibroblasts under hypoxic conditions (DF-Hx) used to test the proliferation of HUVEC and the proangiogenic potential in fibrin gels were assayed in triplicate on human angiogenesis antibody arrays C1000 from RayBiotech. (**A**) The array is divided into two membranes (one represented in the left upper panel, the other in the left lower panel). Proteins detected by the array are indicated on the representation. Representative membranes were shown for DF-Nx medium (center panels) and DF-Hx medium (right panels). (**B**) Results were normalized to the positive control present on each membrane after subtraction of the background. The expression of each protein was compared to the expression of this protein in the DF-Nx condition. Values are average +/− standard deviation.

Proteomic studies were performed to determine the angiogenic mediators’ profiles of conditioned medium from fibroblast cultivated under hypoxic condition vs conditioned medium from fibroblast cultivated under normoxic condition using a macro-array kit. Representative results are shown in Fig. 6A. Individual membranes can be seen in the Supplementary information files 1 to 12. The dashed rectangular boxes highlight the difference between leptin expressions by DF in DF-Nx vs DF in the DF-Hx medium. Results were summarized in Fig. 6B, which represents the ratio between protein expressions in DF-Hx condition and DF-Nx. Because ratio calculation could mask the amount of protein in the conditioned medium, Table 1 is showing the values of the signal for a specific protein compared to the signal of the average of the positive control on the same membrane. Signal intensities of spots are proportional to the relative concentration of the antigen in the sample so they can be compared.

**Table 1:** Protein expression values from human angiogenesis antibody arrays.

	Fold increase	DF-Nx	sd (+/−)	DF-Hx	sd (+/−)	p value
EGF	8,86	0,0056	0,0015	0,0496	0,0031	0,0000
Leptin	5,86	0,0722	0,0187	0,4231	0,0416	0,0000
GM-CSF	4,55	0,0047	0,0002	0,0214	0,0022	0,0092
CXCL5	2,84	0,0637	0,0135	0,1808	0,0114	0,0000
bFGF	2,67	0,0276	0,0017	0,0735	0,0187	0,0015
PlGF	2,24	0,0423	0,0116	0,0949	0,0138	0,3007
IGF-1	1,99	0,0248	0,0043	0,0493	0,0024	0,0006
IL-4	1,71	0,0103	0,0011	0,0176	0,0000	0,2228
MCP-3 (CCL7)	1,55	0,1307	0,0188	0,2030	0,0419	0,0046
IL8	1,37	1,6092	0,0740	2,2012	0,1658	0,0001
CXCL123	1,3	3,3623	0,1755	4,3644	0,2529	0,0000
TIMP-1	1,26	0,4689	0,0361	0,5891	0,0227	0,1068
IL6	1,23	3,9071	0,0814	4,8150	0,0198	0,0000
MMP-1	1,07	0,3044	0,0246	0,3248	0,0202	0,3956
Angiogenin	1,02	0,2275	0,0021	0,2331	0,0231	0,2815
VEGF-A	1	0,2473	0,0183	0,2472	0,0329	0,9957
MCP-1	0,99	0,6679	0,0531	0,6587	0,0389	0,7606
Endostatin	0,93	0,0404	0,0095	0,0378	0,0034	0,3101
TIMP-2	0,92	0,5669	0,0768	0,5220	0,0881	0,4361
Angiostatin	0,9	0,0472	0,0036	0,0426	0,0038	0,2252
I-309 (CCL1)	0,9	0,0171	0,0018	0,0154	0,0007	0,2836
IFNg	0,86	0,0354	0,0005	0,0303	0,0105	0,5991
uPAR	0,82	0,3223	0,0135	0,2662	0,0439	0,0221
TGFb1	0,81	0,0388	0,0030	0,0313	0,0048	0,2851
I-TAC (CXCL11)	0,81	0,0251	0,0030	0,0204	0,0028	0,0817
CCL5	0,8	0,0503	0,0108	0,0403	0,0058	0,9431
IL-1 alpha	0,8	0,0424	0,0016	0,0339	0,0042	0,0059
VEGF R2	0,79	0,0163	0,0008	0,0129	0,0004	0,1818
VEGF R3	0,77	0,0336	0,0067	0,0259	0,0042	0,2033
MCP-4(CCL 13)	0,76	0,0373	0,0011	0,0284	0,0027	0,0267
IL-1 beta	0,75	0,0294	0,0034	0,0222	0,0032	0,0132
Tie-2	0,72	0,0441	0,0009	0,0316	0,0038	0,0008
IL-10	0,71	0,0080	0,0009	0,0057	0,0016	0,1188
MMP-9	0,64	0,0399	0,0025	0,0254	0,0024	0,0059
TPO	0,61	0,1117	0,0080	0,0679	0,0056	0,0387
ANGPT2	0,6	0,3570	0,0373	0,2132	0,0269	0,0000
PECAM-1 (CD31)	0,55	0,0262	0,0002	0,0144	0,0019	0,0000
ANGPT1	0,53	0,4156	0,0420	0,2189	0,0083	0,0028
PDGF-BB	0,3	0,0108	0,0000	0,0032	0,0009	0,3960
VEGF-D	0,21	0,0303	0,0000	0,0064	0,0010	0,0085

As the calculation of the ratio did not allow an estimation of the corresponding amount of each protein, protein expression value normalized to the positive control present on each membrane after subtraction of the background was presented in Table 1. First column: name of the protein, second column: the ratio between signals obtained in DF-Hx condition vs DF-Nx condition. Third column: average value for DF-Nx condition, fourth column: standard deviation for DF-Nx condition, fifth column: average value for DF-Hx condition, sixth column: standard deviation for DF-Hx condition, seventh and last column: p-value using a student t-test between average values from DF-Nx and DF-Hx conditions. Epidermal growth factor (EGF), granulocyte-macrophage colony-stimulating factor (GM-CSF), chemokine (C-X-C motif) ligand **(**CXCL), basic fibroblast growth factor **(**bFGF), insulin-like growth factor 1 (IGF-1), monocyte chemoattractant protein**(**MCP), interleukin-8 (IL-8), Placental growth factor, (PlGF), tissue inhibitors of metalloproteinases (TIMP), Matrix metalloproteinase-1 (MMP-1), vascular endothelial growth factor (VEGF), chemokine (C-C motif) ligand (CCL), Interferon-γ (IFNγ), transforming growth factor-β1 (TGF-β1), VEGF receptor (VEGFR), platelet-derived growth factor-BB (PDGF-BB), Urokinase-type plasminogen activator receptor (uPAR), angiopoietin-1 receptor (Tie2), thrombopoietin (TPO), cluster of differentiation-31 (CD-31).

## Discussion

The link between hypoxia and angiogenesis has been known for a very long time, and sharp scientific pieces of evidence corroborate this link. Based on this mature and robust scientific literature, this study evaluates the potential of medium conditioned by healthy dermal fibroblasts in hypoxia to provide a new cell culture medium, simple to produce and inexpensive, for multiple applications, such as the formation of pseudo-capillary networks by HUVEC in fibrin gels.

This study has shown that fibroblasts under hypoxic condition secrete the soluble factors required to promote the growth and migration of HUVEC (Figs. 1 and 2), followed by the formation and the maturation of capillary-like structures in an *in vitro* fibrin gel model (Figs. 2–4). These structures are longer compared to the ones obtained using a commercially available medium, or medium conditioned by fibroblasts under normoxic conditions. It could also be noted that these structures presented more branching, indicating a higher degree of organization of the capillary-like network.

Most of the commercially available media presently used to cultivate endothelial cells, recreate a proangiogenic context by supplying a basal medium with recombinant proteins such as VEGF, EGF, and bFGF. However, the microenvironment of cells, which requires oxygen and nutrient supply, and then trigger angiogenic signal, is more complicated than just a few factors, even if they are known to be very potent *in vitro*. For example, in our DF-Hx medium, VEGF was present in the same amount as in the DF-Nx medium, but without being competent for the formation and maturation of the capillary-like network. Another point is that if several factors were upregulated after exposure of fibroblasts to hypoxic conditions compared to normoxic conditions (e.g., EGF, leptin, GM-CSF, CXCL5), several others were downregulated (e.g., PDGF-BB, angiopoietin 1 and 2). Fibroblasts under hypoxic conditions secreted the optimal cocktail of factors to revert to the normoxic conditions.

The use of conditioned media to increase angiogenesis in cell culture has also been tested using tumour cells^14^. Nevertheless, the use of media conditioned by healthy fibroblasts under hypoxic conditions presents fewer drawbacks, especially in a clinical translation context. Another study highlighted the benefits of using umbilical cord mesenchymal stem cells conditioned medium^15^. Once again, the use of healthy and well-characterized stromal cells (or even autologous stromal cells when possible) stimulated by hypoxia, seems like a more natural way to produce a proangiogenic cocktail. To support the use of conditioned media from well-characterized cell source, it can be noted that a feeder layer made of irradiated immortalized 3T3 mice cells has been used for a long time (more than 45 years) to culture keratinocytes used in the production of artificial skin^16^. This artificial skin has been grafted in humans, with no adverse effects being reported. Now, to increase the safety of tissue engineering products, this kind of feeder layer has been replaced by irradiated healthy and well-characterized human dermal fibroblasts (^17^). This protocol received agreement from Health Canada, suggesting that the use of conditioned media from healthy and well-characterized dermal fibroblasts will be acceptable in the future.

The most surprising result was the unchanged amount of VEGF-A after exposure to hypoxia. It could result from the length of the 14 days storage period at 4 °C of the conditioned medium before being used (to simulate the actual condition of use in a tissue engineering laboratory) whereas the half-life of VEGF-A in biological fluids is 30 min^18^. Indeed, several technical notes for commercially available VEGF, point out the fact that it should be stored at −20 °C or kept no longer than one week at 4 °C. However, it is also possible that the experimental conditions, cell culture and long term exposure to hypoxic conditions (1 week), did not promote VEGF-A overexpression. An explanation could also be found in a study on the molecular basis of VEGF expression in diabetic tissues^19^. Cell culture was done using DMEM high-glucose, and in this study, fibroblasts from normal foreskin cultured in high glucose medium had a feeble response to hypoxia, especially for the VEGF expression. This decrease in VEGF expression, compared to the same fibroblasts cultured in low glucose, could be due to the partial inhibition by the glycation of interaction between HIF-1a and its coactivator p300. Some studies using mesenchymal stem cells did not obtain the same results (^20^), but this discrepancy could be due to the use of another cell type. However, the results presented in this study can be compared to what was obtained in coculture experiment with wound myofibroblasts in fibrin gels, where better angiogenesis was obtained without an increase in VEGF expression^21^.

Nevertheless, several well-known pro-angiogenic factors, such as EGF and bFGF^22–25^, were overexpressed in the DF-Hx medium. Leptin has been described for its role in connecting obesity and cancer progression. Leptin is closely related to several growth factors, such as VEGF and IGF-1^26^. GM-CSF is also a valuable player to coordinate the role of the different actors of the angiogenic process^27^. CXCL1, 2, 3 and 5 are members of a family of cytokines and are known to play a decisive role in angiogenesis^28,29^. IGF-1 is known to play a role in the stabilization of nascent vessels^30^. Finally, IL-6 and -8 are potent proangiogenic interleukins^31–33^. However, the macro-array used in this study targets proteins related to angiogenesis, and it is possible that essential peptides were not identified at this step. Further proteomic investigations are needed to define the pro-angiogenic factors contained in the DF-Hx medium.

Despite the interest of our study, limitations must nevertheless be noted, which may be the subject of further studies.

First, fibrin gels had been used to recreate angiogenesis for fundamental studies, and their clinical applications mainly remain as a sealant or sutureless graft purposes. This model is very efficient to represent what happens in a fibrin clot and then could mimic surgical glues such as Tisseel, which help control bleeding. It could also be used to improve wound healing in clinical conditions where micro-vascularization is defective, such as diabetic ulcers. Nevertheless, the tissue engineering industry uses many different biomaterials, which present difficulties to be endothelialized, especially large structures and bone repair, and have to be tested. Even if the tissue substitutes produced by the self-assembly techniques can be easily pre-endothelialized and show fast and efficient reperfusion of the graft^34,35^, their production uses expensive commercial media, and it could be interesting to evaluate if DF-Hx medium could replace them. Indeed, the kind of network we obtained was roughly similar to what was previously obtained using the self-assembly protocol to produce engineering tissues (^36–38^). Therefore, we can expect it will be the case with the fibrin gels in case they were used as an implant. The situation of ulcers, and especially diabetic ulcers, is complicated. Diabetes is a multifactorial pathology, and wound healing treatment using pre-endothelialized tissue-engineered graft may fail due to several extrinsic factors. However, the use of a tissue-engineered graft (more complex than just a fibrin gel) was used to successfully treat ulcers^39^. Therefore, it gives reasonable hope that such a strategy could benefit from our study, even if, perhaps, not all the ulcers. One advantage of using a medium helping the formation of the microvascular network before implantation, compared to the inclusion of specific growth factors on the surface of the biomaterials, is the price of the reconstructed tissue, which should remain roughly similar to the one of the non-endothelialized tissue.

Second, to reduce the invasive process of harvesting cells from the patient, using the same stromal cells like the ones used to reconstruct the graft, can be interesting. It should also be an advantage for the clinical translation by avoiding the use of exogenous materials. Thus, we had to explore the potential of the conditioned medium under hypoxic conditions into the various kinds of stromal cells, which are susceptible to be used in tissue engineering such as genito-urinary tissue, breast, heart, and adipose tissues. Other points concerning the cells used are that if HUVEC recreate capillary-like structures roughly similar to the one generated using microvascular endothelial cells (MVEC)^40^, in the context of clinical translation, it could be better to use MVEC from the patient (i.e. not HUVEC). DF-Hx medium potential to support a capillary-like network must also be tested using MVEC. In this context, it is also evident that cells from patients, and not only from healthy volunteers, should be used.

The third limitation is the use of fetal bovine serum in this study. Nevertheless, the use of bovine serum in cell culture, which is the paradigm of an undefined mix, is widely accepted, including for clinical applications. For safety considerations, further studies are nevertheless required to define a cell culture medium exempt from animal proteins.

It must be specified that the hypoxic chamber we used was home-made^6^ and that it required the cells to be taken out to carry out the medium exchanges under a standard culture hood, thus under normoxic conditions. This may explain that the expression of individual factors, well-known to be induced in hypoxic conditions (e.g. VEGF-A), was little or not affected by the conditioning under hypoxia. Nevertheless, a hypoxic workstation is expensive, and its use could be envisaged only if needed. In this study, only conditioning under 2% O_2_ vs 20% O_2_ was tested, as it is well-known that each tissue has a physiologic «normoxia,» which is different^41^. Other oxygen tension should be used, especially for stromal cells, which inhabit tissue where very low oxygen tension exists. Another potentially negative point that could be easily corrected to obtain better results would be to cultivate the cells in the absence of serum, reduce batch to batch variability and limit safety risk^42^, and decrease the non-specific signalling activation of pathways that may interfere with the primary signal.

This study is the first step to establish a robust line of proangiogenic, safe and inexpensive media.

## Materials and methods

### Cell culture

All procedures involving patients were conducted according to the Helsinki Declaration and were approved by the Research Ethical Committee of CHU de Québec-Université Laval (Project 1012–1341). The informed consent of donors for tissue harvesting was obtained for each specimen and experimental procedures were performed in compliance with the CHU de Québec guidelines.

Dermis Fibroblasts (DF) isolation was done as previously described^43^. Briefly, dermis biopsies were collected from the skin of healthy donors undergoing plastic surgeries. After extensive washes in phosphate-buffered saline (PBS) with 100 U/ml penicillin, 25 mg/ml gentamicin and 0.5 mg/ml fungizone (Bristol-Myers Squibb, Montreal, QC, Canada), the skin biopsy were cut into small pieces and incubated in 4-(2-hydroxyéthyl)-1-pipérazine ethane sulphonic acid (Hepes) buffer (pH 7.4; MP Biomedicals, Montreal, QC, Canada) containing 500 mg/ml thermolysin (Sigma) and 1 mM CaCl_2_ (Sigma) overnight at 4 °C. Following incubation, the epithelial layer was mechanically removed from the connective tissue containing fibroblasts (DF). The dermis was transferred in a trypsinization unit containing collagenase H solution (0.125 U/ml; Roche Diagnostics) in Dulbecco–Vogt modification of Eagle’s medium high-glucose (DMEM; Invitrogen, Burlington, Canada), 10% fetal bovine serum (FBS, Hyclone, Logan, UT), 100 U/ml penicillin, 25 mg/ml gentamicin and sodium bicarbonate (DF medium). After 4 h of incubation at 37 °C, cells were harvested by centrifugation at 300 g for 10 min. DFs were then seeded into culture flasks with this medium and incubated at 37 °C in a humidified 8% CO_2_ atmosphere. The medium was exchanged three times a week. Passages were performed when DFs reached 80–90% confluence, using trypsin (0.05% trypsin: ICN Biomedicals, Irvine, CA, USA; 0.01% EDTA: J.T. Baker, Phillipsburg, NJ, USA). For the irradiated pool of DFs (not the one used to condition the media), cells at passage 3 were cultured until they reached 80–90% confluence into 75 cm^2^ culture flasks and exposed to 60 Gy gamma irradiation from a 60 Ci source. DFs were irradiated to inhibit their proliferation for the subsequent fibrin gel experiments^21^.

Human umbilical vein endothelial cell (HUVEC) isolation was performed as previously described^44^. Briefly, HUVECs were obtained from healthy newborn umbilical veins by enzymatic digestion with 0.250 mg/mL thermolysin. Veins were cannulated at both ends and washed with calcium-free Hepes solution. A thermolysin solution was then injected to rinse and fill the vein, and the cord was placed in calcium-free Hepes at 37 °C. After 30 minutes of incubation, the veins were perfused with M199 medium (Sigma) containing 10% FetalClone II (Hyclone) and antibiotics (100 U/mL of penicillin G and 25 mg/mL of gentamicin) at 37 °C. HUVECs were centrifuged and resuspended in M199 medium supplemented with 20% newborn calf serum (NCS), 2.28 mM glutamine (Invitrogen), 40 IU/mL heparin (Leo Laboratories, Pickering, Canada), 20 mg/ml endothelial cell growth supplement (ECGS, Sigma), and antibiotics (100 U/mL of penicillin G and 25 mg/mL of gentamicin). Cells were incubated at 37 °C in a humidified 8% CO_2_ atmosphere. The medium was exchanged three times a week. At passage 2, HUVECs were centrifuged and suspended in EBM-2 medium (Clonetics, San Diego, CA, USA) containing fetal bovine serum (FBS) 2%, 100 U/mL of penicillin G, and 25 mg/mL of gentamicin, vascular endothelial growth factor (VEGF), recombinant human Fibroblast growth factor, Basic (rhFGF-B), recombinant human Epidermal growth factor (rhEGF), insulin, and ascorbic acid. This culture medium will be abbreviated as EGM-2MV. Cells were incubated at 37 °C in a humidified 8% CO_2_ atmosphere and passaged at 80–90% confluence. The medium was exchanged three times a week.

### Hypoxic chamber and cell culture

A desiccator cabinet (Nalgene, Rochester, NY, USA) was modified to include two holes that were drilled on top of the cabinets: one at the end to the right and one in front to the left. Adapters were placed in these holes, allowing the insertion of valves to control O_2_ flow. O-rings assured the tightness on each side of the wall. Hypoxic chambers were installed in a standard cell culture incubator at 37 °C. Gas cylinder (Linde Canada, Quebec City, QC, Canada) containing O_2_-reduced gas mixes such as 2% O_2_, 8% CO_2_ and 90% N_2_ was connected to the chamber. After each chamber opening, the atmosphere was renewed with an appropriate O_2_-reduced gas mix. During the gas exchange, a pipe evacuates the old gas outside of the incubator. O_2_ pressure was verified using a Pac 5500 O2 (Dräger Safety, Pittsburgh, PA, USA). The experimental atmosphere was humidified using a Petri dish with apyrogene water that was added to the bottom of the chamber. DF at passage 3 were cultivated into 75 cm^2^ culture flasks with 10 ml of DF medium (i.e. DMEM, 10% FBS and antibiotics) in normoxic condition until they reach confluence. Half of the flasks were then transferred into hypoxic chambers for 1, 2 or 3 weeks, whereas the remaining flasks stayed in culture under normoxic atmosphere for the same duration as the hypoxia-exposed ones. Media were harvested three times a week and replaced by new media. The harvested media for each condition were pooled by week (at weeks 1, 2 and 3) before to be stored at -80 °C. Aliquots were stored apart for the measurement of cell proliferation, fibrin gel experiments, and proteomic analyses.

### Doubling time measurement

HUVECs were seeded at 10% confluence in 12-well plates. Appropriate medium (DF medium as a negative control, DF-Nx medium for DF medium conditioned by DFs under the normoxic condition and DF-Hx medium for DF medium conditioned by DFs under hypoxic condition) was exchanged daily for fresh medium for four days. Each day, HUVECs from three wells were harvested and numbered separately using a Coulter-Beckmann Z2 system. A proliferation curve was performed to calculate doubling time. The regression curve formula extrapolated from the proliferation curve was P = Po e^gx^, where P was the number of cells on day x, Po was the cell number at day 0 and g the exponential coefficient. Doubling time was calculated with the following formula: D = ln2/g.

### Three-dimensional fibrin gels

HUVECs and irradiated DFs were trypsin-treated, harvested and resuspended in DMEM without serum at a concentration of 0.8 10^6^ cells/ml. Fibrin gels were immediately prepared using 200 µl bovine plasma fibrinogen (Sigma-Aldrich) at 16 mg/ml combined with 10 µl bovine plasma thrombin (Sigma-Aldrich) at 40U/ml (each solution diluted into DMEM without serum) and 100 µl of each cell suspension^21^. This mix was poured into each well of 24-well plates corresponding to 8 mg of fibrinogen, 1 U of thrombin and 200,000 cells of each type: HUVECs and irradiated DFs. After a 30 min incubation, polymerized fibrin gels were covered with 1 ml of EGM-2MV as a positive control, DF medium as a negative control, DF-Nx medium, and DF-Hx medium. Fibrin gels were maintained in culture for 14 days. Media were changed three times a week. Fibrin gels were washed with PBS and fixed in 3.7% formol for histological analysis.

### Histology

Formol-fixed fibrin gels were processed for immunofluorescence (IF). They were incubated overnight with a primary antibody, CD31 (AF806, R&D Systems, Minneapolis, MN) in 1% bovine serum albumin–phosphate-buffered saline before a 45 min incubation with a secondary antibody conjugated with Alexa-594. As a negative control, the primary antibody was omitted. Nuclei were counterstained with Hoechst reagent. Gels were viewed using an Axio Imager M2 microscope (Carl Zeiss).

### Proteomic analyses

DF medium (2 aliquots), DF-Nx (3 aliquots) and DF-Hx (3 aliquots) were analyzed using the RayBio C-Series Human Angiogenesis Antibody Array 1000 Kit. A combination of human angiogenesis antibody array C1 & C2 detects 43 human angiogenic factors. Kits were used following the manufacturer’s instruction. Signals from membranes were detected using a Fusion Fx7 (Vilbert-Lourmat, Marne-La-Vallée, France). The signal detected by this apparatus remained linear in a range of 64 000 from the weakest to the strongest signal. These features limited errors in quantitation. Signals were then treated by ImageJ software (NIH, Bethesda, MD, USA). Values from the blank were substracted to all values and data normalized using the positive control on each membrane. Data were reported as the ratio between the signal generated by the conditioned medium under the hypoxic condition to the signal generated by the conditioned medium under normoxic condition. More precisely, individual values were normalized to the value of the positive control for each membrane after subtraction of the value of the negative control. V = (Vx − V_N_)/(V_P_ − V_N_) where V is the normalized value, Vx the raw data, V_N_ the average value of the negative controls (n = 6 per membrane) and V_P_ the average value of the positive controls (n = 6 per membrane) and, finally, ratio R = V(DF-Hx)/V(DF-Nx).

### Statistical analysis

Data are expressed as the mean ± standard deviation. ANOVA test was performed, followed by a Student’s t-test (which was used to compare two data sets). The level of significance was established at P ≤ 0.05. Unless specifically mentioned, all experiments were done in triplicate. All statistics were performed using Microsoft Office Excel and its analysis ‘ToolPak’ add-in program.

## Supplementary information

Supplementary information.

Supplementary information 2.

Supplementary information 3.

Supplementary information 4.

Supplementary information 5.

Supplementary information 6.

Supplementary information 7.

Supplementary information 8.

Supplementary information 9.

Supplementary information 10.

Supplementary information 11.

Supplementary information 12.

## Data Availability

The datasets used and/or analyzed during the current study are available from the corresponding authors on reasonable request.
