# Supplementary material for: Conditioned medium produced by fibroblasts cultured in low oxygen pressure allows the formation of highly structured capillary-like networks in fibrin gels
Source: Sci Rep. 2020 Jun 9;10:9291. doi: 10.1038/s41598-020-66145-z (PMC7283357; doi:10.1038/s41598-020-66145-z)
Supplement: Supplementary file 12 — Supplementary information 12. [file 41598_2020_66145_MOESM12_ESM.docx]

**Original papers (research article)**

**Manuscript Title:**

**Conditioned medium produced by fibroblasts cultured in low oxygen pressure allows formation of highly structured capillary-like networks in fibrin gels.**

***Short Title: conditioned medium in hypoxia allows angiogenesis in fibrin gels.***

**Authors:** Christophe Caneparo M.Sc. ^1^, Clément Baratange M.Sc. ^1,2,§^, Stéphane Chabaud, Ph.D. ^1,*^, Stéphane Bolduc, M.D. ^1, 3,*^

**SUPPLEMENTARY DATA**

**Photographs of membranes from RayBio C-series Human angiogenesis Antibody Array C1000.**

Nomenclature: Fig.S**X** aah-ang-Y-Zsec; X is the number of the supplementary figure, y is the identification of the membrane (1 for C1, 2 for C2) and Z is the exposure time. The first upper three membranes are incubated with DF-Nx, the first lower three membranes are incubated with DF-Hx the two last membranes (upper and lower) are incubated with non-conditioned media.

Fig.S1 aah-ang-1-8sec

Fig.S2 aah-ang-1-15sec

Fig.S3 aah-ang-1-30sec

Fig.S4 aah-ang-1-60sec

Fig.S5 aah-ang-1-120sec

Fig.S6 aah-ang-1-240sec

Fig.S7 aah-ang-2-10sec

Fig.S8 aah-ang-2-30sec

Fig.S9 aah-ang-2-60sec

Fig.S10 aah-ang-2-120sec

Fig.S11 aah-ang-2-240sec
